# FGF2 mediates DNA repair in epidermoid carcinoma cells exposed to ionizing radiation

**DOI:** 10.3109/09553002.2012.706358

**Published:** 2012-07-20

**Authors:** Mélanie Marie, Sophia Hafner, Sandra Moratille, Pierre Vaigot, Solène Mine, Odile Rigaud, Michèle T. Martin

**Affiliations:** ^1^CEA, iRCM, Laboratoire de Génomique et Radiobiologie de la Kératinopoïèse, Evry, France; ^2^BASF Beauty Care Solutions France SAS, Laboratoires Sérobiologiques, Pulnoy, France

**Keywords:** A431 carcinoma cell line, cancer stem cells, side population, ionizing radiation, DNA repair, FGF2

## Abstract

**Purpose:**

Fibroblast growth factor 2 (FGF2) is a well-known survival factor. However, its role in DNA repair is poorly documented. The present study was designed to investigate in epidermoid carcinoma cells the potential role of FGF2 in DNA repair.

**Materials and methods:**

The side population (SP) with cancer stem cell-like properties and the main population (MP) were isolated from human A431 squamous carcinoma cells. Radiation-induced DNA damage and repair were assessed using the alkaline comet assay. FGF2 expression was quantified by enzyme linked immunosorbent assay (ELISA).

**Results:**

SP cells exhibited rapid repair of radiation induced DNA damage and a high constitutive level of nuclear FGF2. Blocking FGF2 signaling abrogated the rapid DNA repair. In contrast, in MP cells, a slower repair of damage was associated with low basal expression of FGF2. Moreover, the addition of exogenous FGF2 accelerated DNA repair in MP cells. When irradiated, SP cells secreted FGF2, whereas MP cells did not.

**Conclusions:**

FGF2 was found to mediate DNA repair in epidermoid carcinoma cells. We postulate that carcinoma stem cells would be intrinsically primed to rapidly repair DNA damage by a high constitutive level of nuclear FGF2. In contrast, the main population with a low FGF2 content exhibits a lower repair rate which can be increased by exogenous FGF2.

## Introduction

Many malignant tumours are composed of morphologically and phenotypically heterogeneous cell populations, with varying self-renewal capacities, degrees of differentiation, and tumorigenic potentials. These observations have led to the development of the theory which posits that neoplasms can be hierarchically organized and contain cancer stem cells (CSC). Cancer stem cells are defined as cells within a tumour which have the capacity to self-renew and generate the heterogeneous lineages of cancer cells that comprise the tumour (Clarke et al. 2006). This definition implies that anticancer therapy can eliminate a tumour only if all cancer stem cells are killed. In the context of radiotherapy, it is thus crucial to characterize the different subpopulations found in a tumour, as well as all their strategies to resist to radiation stress (Baumann et al. 2008, 2009).

A possible mechanism of CSC radioresistance is a more active repair of DNA damage, as illustrated in different models, including glioblastoma (Bao et al. 2006) or breast cancer (Zhang et al. 2010b). Another mechanism of radioresistance of CSC is the constitutive activation of signalling pathways (Zhou and Zhang 2008). The fibroblast growth factor 2 (FGF2) and its intracellular signalling proteins are known to act in a survival pathway protecting cells from radiation-induced cell death (Paris et al. 2001, Zhang et al. 2010a). A new role for FGF2 has recently been described, which is the activation of DNA repair in HeLa cells (Ader et al. 2002) and in human epidermal stem cells (Rachidi et al. 2007, Harfouche et al. 2010). The present study aimed at investigating such a role in epidermoid carcinoma stem cells exposed to γ-rays.

Several FGF2 isoforms have been described, which are generated from a single gene by alternative initiation of translation on a common mRNA. The 18 kDa isoform is secreted and acting through binding to specific transmembrane receptors which activate intracellular signalling proteins, including the mitogen-activated protein kinase kinases (MEK). The other forms with higher molecular weights (HMW) are predominantly located in the cell nucleus and modulate numerous cellular activities by intracrine mechanisms (Delrieu 2000, Sorensen et al. 2006). Members of the FGF2 canonical signalling cascade, initially described in the membrane and cytoplasm, have also recently been found operating in the nucleus (Mikula and Bomsztyk 2011).

There is evidence that squamous cell carcinoma are maintained by cancer stem cells (Prince and Ailles 2008, Richard and Pillai 2010). In the present study, carcinoma stem cells are modelled in the human A431 cell line. This cell line is known for its high radioresistance (Pekkola-Heino et al. 1989) and for its capacity to generate carcinoma with high proliferation rate and poor differentiation. The selection of the cell population which is able to reinitiate a tumour can be achieved by several strategies, including cell sorting on specific membrane markers and the cell capacity to grow as spheres (Zhou and Zhang 2008). Another widely used method is stem cell sorting based on their capacity to exclude the Hoechst 33342 dye through active membrane transporters (Richard and Pillai 2010, Golebiewska et al. 2011). This method is efficient for A431 cells (Bortolomai et al. 2010), as the side population (SP cells) exhibits CSC properties and generates carcinoma in immunodeficient mice (Geng et al. 2011).

In the present study, we characterized DNA repair and FGF2 expression in SP cells isolated from A431 cells and compared them to the main population of carcinoma cells (MP cells). We found that SP cells repaired more rapidly radiation-induced damage than MP cells, and that the FGF2 pathway could mediate this rapid repair.

## Materials and methods

### Cell culture and gamma irradiation

A431 cells, isolated from the vulvar squamous cell carcinoma from an 85-year-old patient, were obtained from the American Type Culture Collection (ATCC, Manassas, VA, USA) and maintained in Dulbecco's modified eagle's medium (DMEM) Glutamax (Invitrogen, Carlsbad, CA, USA) supplemented with 10% Fetal Bovine Serum (FBS, Hyclone, Logan, UT, USA), 100 U/ml penicillin and 100 *μ*g/ml streptomycin (Invitrogen) in a humidified incubator at 37°C in 5% CO_2_. Cells were passaged at 80% confluence using 1 × Trypsin ethylenediaminetetraacetic acid (EDTA, Invitrogen). Cells were irradiated with 2 or 4 Gy at a dose rate of 0.93 Gy/min using a cesium-137 γ-rays source (IBL 637, CIS Bio International, Saclay, France).

### Isolation of SP cells

Sorting of SP cells was based on their specific ability to efflux Hoechst 33342 DNA dye. In all experiments, dye staining and cell sorting were performed when cell cultures reached 50% confluence. Briefly, cells were harvested and resuspended in DMEM containing 2% FBS and 6 *μ*g/ml of Hoechst 33342 (Sigma-Aldrich, St Louis, MO, USA). Cells were then incubated for 1 h at 37°C under shaking. Flow cytometry analysis and cell sorting were performed on a MoFlo cell sorter (Beckman Coulter, Brea, CA, USA). Two cell populations were sorted: SP cells, that efficiently excluded the Hoechst dye and represented a distinct and small fraction of weakly fluorescent cells, and MP cells, that showed high levels of Hoechst red and blue fluorescences. For specific inhibition of the dye exclusion, verapamil (250 *μ*M, Sigma-Aldrich) was added during the incubation process with Hoechst 33342.

### Alkaline comet assay

Quantification of DNA damage was performed using the alkaline comet assay, which allows the detection of single and double-strand breaks, as well as alkali-labile lesions in individual cells (Olive 1999, Rigaud et al. 2010). Comet analysis was performed using the image analysis Komet 5.5 software (Andor Technology, Belfast, Northern Ireland, UK). The Olive Tail Moment (OTM), defined as the product of the tail length by the ratio of the fluorescence intensity between the head and the tail, was taken as the index of damage. For each treatment, the average tail moment was determined from the analysis of 200 comets per sample and each experiment was done in triplicate. In experiments designed to investigate the involvement of the FGF2 pathway in DNA repair, UO126 inhibitor (5 μg/ml, Millipore, Billerica, MA, USA) was added 30 min before radiation exposure. To study the effects of exogenous FGF2 supplementation on DNA repair, human recombinant basic FGF (FGF2, 10 and 50 ng/ml; Peprotech, Rocky Hill, NJ, USA) was added to the growth medium 1 h before radiation exposure and subsequent DNA repair assessment.

### FGF2 enzyme-linked solid phase immunosorbent assay

The FGF2 enzyme linked immunosorbent assay (ELISA) kit (R&D Systems, Minneapolis, MN, USA) was used to quantify the level of FGF2 protein. For total intracellular FGF2 protein, cells were harvested in radioimmunoprecipitation assay buffer (RIPA) protein lysis buffer (Sigma-Aldrich). For nuclear and cytoplasmic FGF2, protein extracts were prepared as described by Schreiber et al. (Schreiber et al. 1989). Total cell protein contents were determined using a micro BCA Protein Assay Kit (Thermo Scientific, Rockford, IL, USA). To quantify secreted FGF2 protein, the cell culture medium was collected 48 h after irradiation. The FGF2 protein level is expressed as pg of FGF2 per 100 *μ*g of total proteins.

### Quantitative RT-PCR

Total RNA was extracted using the RNeasy Micro kit (Qiagen, Venlo, The Netherlands). Reverse transcription polymerase chain reaction (RT-PCR) was performed as previously described (Harfouche et al. 2010). Amplification was carried out using FGF2 forward (5′CGACCCTCACATCAAGCTACAA3′) and reverse (5′GCA CACACTCCTTTGATAGACACAA3′) primers. Data were normalized to the 18S RNA which was simultaneously amplified using 18S forward (5′CGATGCGGCGGCGTTATT3′) and reverse (5′CCTGGTGGTGCCCTTCCGT3′) primers.

### Statistical analysis

Student's *t*-test was used to assess statistically significant differences at *p* < 0.05. Each experiment was performed at least in triplicate.

## Results

**Figure 1.**
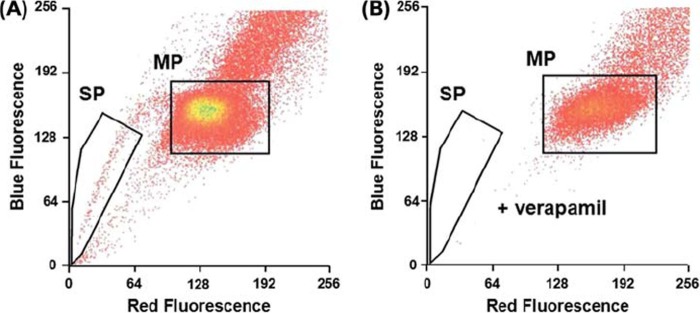
A side population (SP cells) was isolated from the A431 carcinoma cell line. SP cells representing 1.6% (*n* = 20) of total cells were isolated from the A431 carcinoma cells using the Hoechst 33342 dye efflux method (A). An inhibitor of Hoechst efflux (verapamil, 250 *μ*M) was used to check specificity of the SP phenotype (B). SP, side population; MP, main population.

A side population can be isolated from the carcinoma cell line. A subpopulation representing 1.6% (± 0.44) of the total A431 carcinoma cells was identified as SP cells by its capacity of rapid Hoechst dye exclusion (Figure 1A) and its response to the ATP-binding cassette pump (ABC pump) inhibitor verapamil (Figure 1B). MP cells were defined as the main population that cannot exclude Hoechst dye. Sorting gates for SP cells and MP cells were defined as illustrated in Figure 1A.

### Fast repair of radiation-induced DNA damage in SP cells

**Figure 2.**
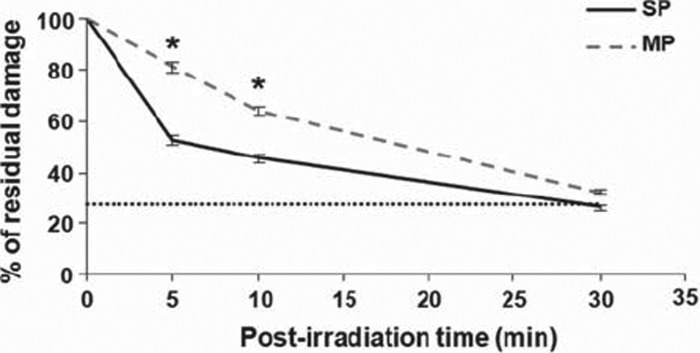
Fast repair of radiation-induced DNA damage in SP cells. Following gamma irradiation (4 Gy), SP cells exhibited a faster repair of DNA damage than MP cells as measured by the alkaline comet assay. The level of damage in non-irradiated cells (dotted line) was similar for both cell populations. Data are the mean values + / − SEM (standard error of the mean) of three independent experiments. Asterisks indicate a significant difference (*p* < 0.05). SP, side population; MP, main population.

The alkaline comet assay was used to quantify global DNA damage including single-, double-strand breaks and alkali-labile lesions in irradiated cells (4 Gy). Ionizing radiation induced similar levels of DNA damage in both cell populations (OTM: 11.0 ± 2.0 SP cells; 11.4 ± 2.4 MP cells). This damage was then more rapidly repaired in SP cells than in MP cells, with 50% of the damage repaired within 5 minutes in SP cells, as compared to 20% in MP cells (Figure 2). Dye accumulation does not explain the slower repair rate in MP cells, as similar repair kinetics were obtained with unlabelled cells (data not shown).

### The FGF2 pathway mediates rapid DNA repair

**Figure 3.**
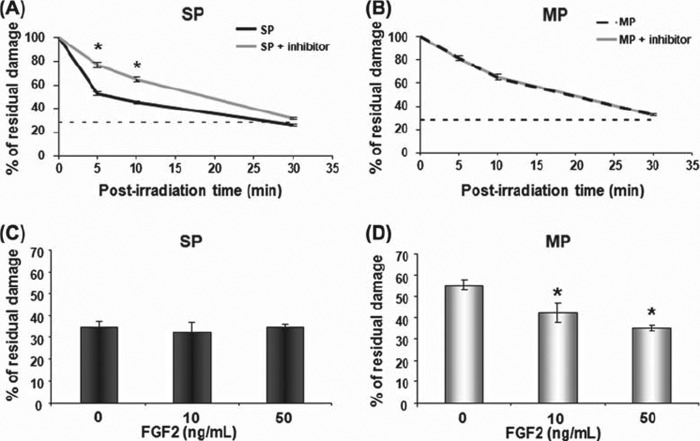
The FGF2 pathway regulates DNA repair. Addition of UO126 inhibitor (5 *μ*g/ml) 30 min before radiation exposure slowed down DNA repair in SP cells (A), whereas DNA repair remained unaltered in MP cells (B). Addition of exogenous FGF2 (10 and 50 ng/ml) 1 h before radiation exposure accelerated DNA repair only in MP cells (C and D, alkaline comet assay at 10 min). Data are the mean values + / − SEM (standard error of the mean) of three independent experiments. Asterisk indicates a significant difference (*p* < 0.05). SP, side population; MP, main population.

As FGF2 cell signalling has been involved in DNA repair of normal epidermal stem cells (Harfouche et al. 2010), such a role was investigated in epidermoid carcinoma stem cells. The FGF2 signalling pathway was blocked at the level of the mitogen-activated protein kinase kinase 1/2 (MEK1/2), using the UO126 inhibitor. When treated with UO126 for 30 min before irradiation, SP cells lost their capacity of rapid DNA repair (Figure 3A), whereas the inhibitor had no effect on MP cells (Figure 3B). On the other hand, exogenous FGF2 added to the cell culture medium accelerated DNA repair in MP cells in a dose-dependent manner (Figure 3D), but had no effect on SP cells (Figure 3C). These data showed that FGF2 signalling could modulate DNA repair in carcinoma cells, via endogenous or exogenous FGF2.

### SP cells express a high constitutive level of FGF2

**Figure 4.**
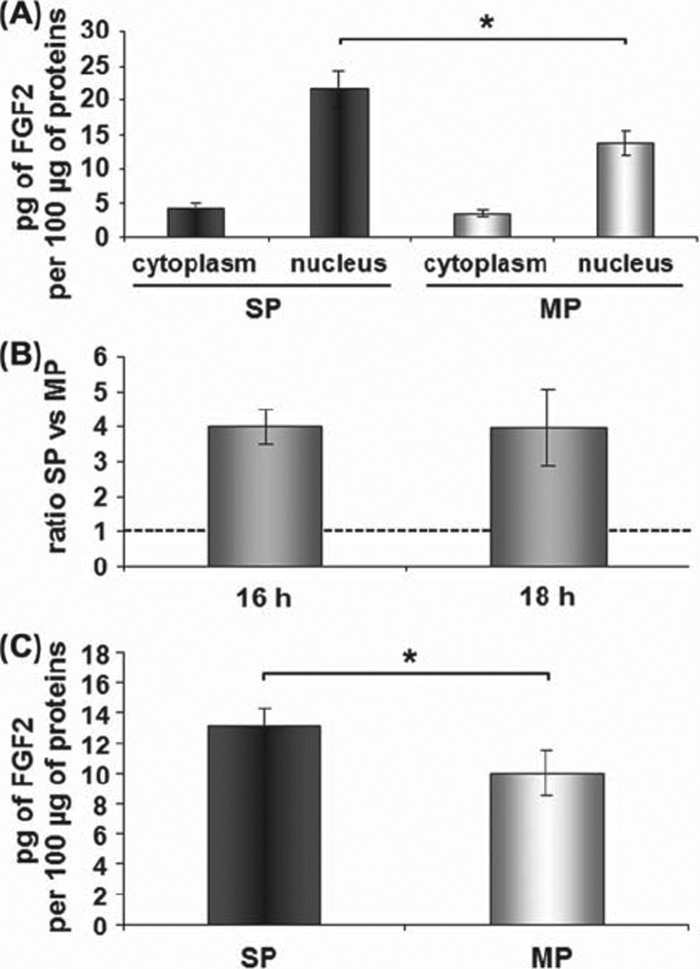
High constitutive intracellular FGF2 in SP cells. Immediately after cell sorting, quantification of nuclear and cytoplasmic contents of FGF2 protein by ELISA showed that SP cells contained more nuclear FGF2 than MP cells (A). After overnight culture, FGF2 mRNA was 4-fold higher in SP cells than in MP cells (B) and the total intracellular content of FGF2 protein was higher in SP than in MP cells (C). Data are the mean + / − SEM (standard error of the mean) of three (A and B) or four (C) independent experiments. Asterisks indicate a significant difference (*p* < 0.05). SP, side population; MP, main population.

To investigate FGF2 expression, the contents of the nuclear and cytoplasmic growth factor were quantified immediately after cell sorting. SP cells had a higher intracellular content of FGF2 protein than MP cells, due to a higher level in the nuclear fraction (Figure 4A). After an overnight culture, the constitutive level of FGF2 remained higher in SP than in MP cells at both the mRNA (with a 4-fold increase in SP versus MP cells, Figure 4B) and intracellular protein level (Figure 4C).

### Ionizing radiation induces FGF2 secretion by SP cells

**Figure 5.**
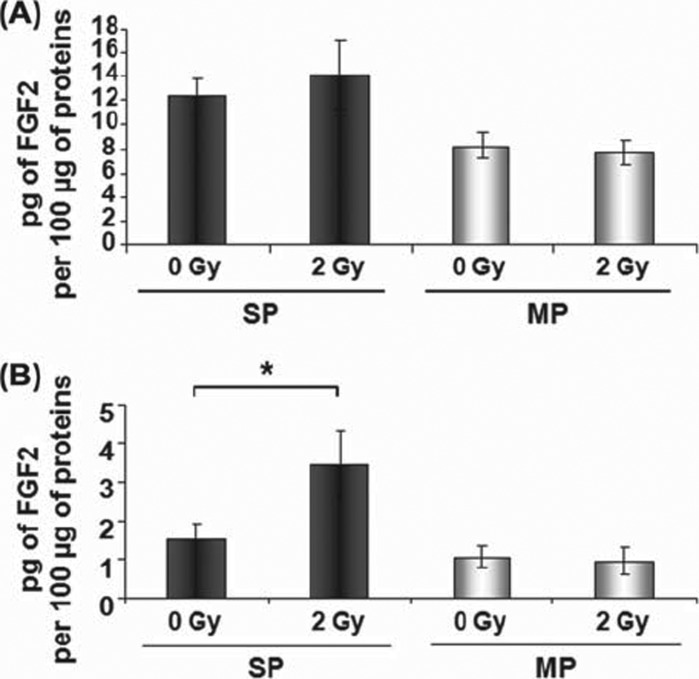
γ-rays induce FGF2 secretion by SP cells. Intracellular and secreted FGF2 were quantified by ELISA. After a culture period of 48 h, the total intracellular content of FGF2 protein remained higher in SP than in MP cells and was not altered after 2 Gy in both cell populations (A). Only SP cells secreted FGF2 in cell culture medium 48 h after irradiation with 2 Gy (B). Data are the mean values + / − SEM (standard error of the mean) of three (A) or five (B) independent experiments. Asterisk indicates a significant difference (*p* < 0.05). SP, side population; MP, main population.

To investigate the radiation effects on intracellular and secreted levels of FGF2, cells were irradiated with 2 Gy after an overnight culture following cell sorting. The cell culture medium was harvested and whole cell protein extracts were prepared 48 h after irradiation. The intracellular contents of FGF2 in both SP and MP cells were not altered 48 h after irradiation (Figure 5A), whereas irradiation induced FGF2 secretion only in SP cells (Figure 5B).

## Discussion

FGF2 is known to be a wide-spectrum survival factor, promoting cell resistance in irradiated normal tissues (Zhang et al. 2010a), notably through the down regulation of apoptosis (Fuks et al. 1994). Its role in the resistance of tumour cells to ionizing radiation has also been shown (Cohen-Jonathan et al. 1997). However, the role of FGF2 in DNA repair has been poorly documented (Ader et al. 2002, Harfouche et al. 2010). We here address the potential role of FGF2 in DNA repair in the A431 carcinoma cells (MP cells) and in a small sub-population which expresses characteristics of cancer stem cells (SP cells).

We found that SP cells were characterized by specific FGF2 expression and activities, which differ from the main population of carcinoma cells. Firstly, SP cells constitutively expressed a higher level of FGF2, predominantly in the nuclear fraction. Secondly, when FGF2 signalling was inhibited in SP cells, DNA repair was slowed down. On the other hand, SP cells responded to DNA damage by secreting FGF2. The present data support the view that carcinoma stem cells are intrinsically primed to rapidly repair DNA damage, notably through high constitutive nuclear level of FGF2, and could sustain late protective responses through the stress-induced secreted form of the factor.

On the contrary, MP cells expressed constitutively a lower level of nuclear FGF2 and did not secrete the factor after irradiation. DNA repair of radiation-induced damage was stimulated by addition of exogenous 18 kDa isoform of FGF2. The occurrence of such a repair regulation in a tumour environment is an open question. Secreted FGF2 can be released by several cell types around a tumour, mainly from the stroma and vasculature. For skin carcinoma, cells from the dermis, fibroblasts and endothelial cells, can be the main sources of secreted FGF2.

Several FGF2 isoforms have been described. Only the 18 kDa isoform can be secreted in the medium, where it acts as a conventional growth factor, through binding to cell-surface receptors and activation of intracellular signalling proteins. The four FGF2 isoforms with high molecular weight (22, 22.5, 24 and 34 kDa) are not secreted, but are translocated to the nucleus via nuclear localization sequences, and then regulate cell growth and behavior through intracrine mechanisms (Okada-Ban et al. 1999, Sorensen et al. 2006). A study performed in HeLa 3A carcinoma cells has shown that overexpression of the nuclear 24 kDa isoform using DNA vectors induces increased repair of radio-induced double-strand breaks together with activation of the DNA-dependent protein kinase (DNA-PK) repair enzyme (Ader et al. 2002). The authors proposed the 24 kDa FGF2 isoform as a new regulator of DNA repair in cancer cells. In the present study, we propose that the fast repair in carcinoma stem cells could be mediated by the intracellular HMW isoforms of FGF2, in an intracrine manner. The assessment of the different nuclear isoforms would be necessary to define the downstream effectors involved in the regulation of DNA repair, but this assessment is currently hindered by the lack of specific antibodies. On the other hand, the alkaline comet assay used in the present study is known to mainly evaluate single-strand breaks repair, which occurs during the first hours of repair kinetics. The assessment of repair of double-strand breaks, which are less abundant and follow a long repair kinetics, would be complementary and of great interest, owing to the deleterious effects of these lesions.

The present study and the data reported by Ader et al. (2002) could open a possible new way to sensitize cancer cells to radiotherapy. Although targeting FGF2 signalling as a cancer therapy has been lagging behind that of other receptor tyrosine kinases, including epidermal growth factor (EGF) signalling, clinical reagents that specifically target the FGFs or FGF receptors are being developed and included in clinical trials (Turner and Grose 2010). Data presented here also support the hypothesis that inhibition of the canonical FGF2 pathway could potentiate radiotherapy.

In conclusion, FGF2 appears to be an activator of DNA repair in carcinoma cells. This function appears to be constitutively operating in SP cells, because of a high endogenous nuclear FGF2 content.
